# Impact of amount of straw on pig and pen hygiene in partly slatted flooring systems

**DOI:** 10.1186/s12917-020-02594-y

**Published:** 2020-10-07

**Authors:** Torun Wallgren, Nils Lundeheim, Stefan Gunnarsson

**Affiliations:** 1 Swedish University of Agricultural Sciences (SLU), Department of Animal Environment and Health, P.O. Box 7068, S-75007 Uppsala, Sweden; 2 Swedish University of Agricultural Sciences (SLU), Department of Animal Breeding and Genetics, P.O. Box 7054, S-75007 Uppsala, Sweden; 3 Swedish University of Agricultural Sciences (SLU), Department of Animal Environment and Health, P.O. Box 234, S-53223 Skara, Sweden

**Keywords:** Grower, Finisher, Fattening pig, Enrichment

## Abstract

**Background:**

Straw is a beneficial enrichment material for pigs, shown to improve welfare through reducing tail biting. Straw has previously been identified as one of the means of how to raise pigs without tail docking, through improving natural exploratory behaviour. Straw has however been linked to poor pen hygiene, making farmers reluctant to use straw and has largely not been implemented in commercial farming worldwide. Straw is a beneficial enrichment material for pigs, shown to improve welfare and reduce abnormal behaviour such as tail biting.

**Results:**

This study investigates the impact of straw on pig and pen hygiene in pens with partly slatted floor in three grower and four finishing pig batches on five commercial farms (2329 pigs, 211 pens) in Sweden which were providing straw daily. Each batch was divided into two treatments; Control: 50-600 g straw/pen/day based on the farm normal straw ration; and Extra straw; (=doubled Control ration). The pens were scored based on cleanliness of the pigs, solid and slatted pen floor every second week. The pig and pen hygiene were mostly scored as clean in both treatments, overall around 1% of the observations were considered dirty/soiled.

**Conclusions:**

As very few pens or pigs were considered dirty, it was concluded that straw provision is possible without risking poor pig and pen hygiene. Few observations in this study were considered dirty regardless of amount of straw that was provided, and had likely to do with other factors in the production rather than straw ration. These results implies that straw could be used in partly slatted pens in order to improve pig environment but more research is needed to quantify the impact of other external factors related to climate (e.g. temperature, humidity, velocity).

## Background

Over 90% of EU pigs are tail docked to minimise tail biting which causes reduced welfare and production [1]. Although tail biting is a multifactorial issue, lack of long straw was identified as the largest risk factor for tail biting through an EFSA report on the subject [2]. Several studies have investigated the effect of straw amount on the level of tail biting on both research farms [3, 4] and in commercial settings [5, 6]. Actual implementation of straw has however largely not taken place and there are still lack of management routines on how to provide straw.

One reason why straw provision has not been implemented is the farmer opinion that straw may block the slatted flooring, manure handling system and stack mired straw in pens, disturbing the pen environment [7–9]. In a survey among Swedish farmers using straw it was considered that blockage of slats and manure handling was the most common reason for not increasing straw rations although problems in the manure handling system were quite rarely reported [6]. Furthermore, in practice straw was not reported to be associated with increased need of manual cleaning [5] which has previously been proposed by e.g. Tuyttens [10]. Previous studies show that pig hygiene was worse in straw-based compared to fully slatted systems [11]. However, fully slatted pens substantially reduce the possibility to provide straw on the floor since most straw will quickly pass through the slats and reduces the behavioural benefits for the pigs. Pigs in straw-based systems may purposely soil their lying area to enabling wallowing during hot conditions, which may impair pig hygiene [11] but may also be an indication of poor indoor environment.

There is lack of knowledge of how straw provision impact hygiene under commercial conditions. The aim of this study was to investigate the pig and pen hygiene in systems with partly slatted flooring in farms that are providing increased straw ration compared to standard level. It was further hypothesised that poor pen hygiene would subsequently lead to poor pig hygiene, as pigs will be forced to lie down on soiled flooring. Poor hygiene on the solid floor will have larger impact on the pig hygiene while poor hygiene on the slatted floor will have less impact as long as the pigs are not forced to lie on the slatted floor due to space limitations. The gained knowledge may be used to facilitate farmers in conducting informed decisions regarding straw usage in commercial production in partly slatted flooring systems.

## Results

### Pig hygiene

#### Grower level

In grower pens, 99.4% of the observed pigs were scored 1 (ranging from 1 to 2 in C and 1–3 in ES treatment); 99.1% in C and 99.5% in ES treatment. On farm G1 scores ranged from 1 to 2 in both treatments, on farm G2 scores ranged from 1 to 2 in C treatment and 1–3 in ES treatment, on G4 all pigs were scored 1 at all occasions. In total, 0.8% of the observations on G1 and 1.1% on G2 were considered dirty.

No significant treatment effect on pig hygiene was found on any farm on grower level (Table 1).

**Table 1:** Impact of treatment on pen and pig hygiene scores. The table displays where Treatment had an effect on hygiene. For the full table, see Appendix I. Identical Farm number indicates that the farm produced both grower and finisher pigs. WIP indicates Weeks in Production

			Slatted floor area hygiene			Solid floor area hygiene			Pig Hygiene		
**Farm**	**Treatment**	**WIP**	**Clean**	**Not Clean**	***p*-value**	**Clean**	**Not Clean**	***p*-value**	**Clean**	**Not Clean**	***p*-value**
**G2**	**C**	**3**	14	10		24	0		253	0	
	**ES**		20	0	0.0009	20	0	N.E.	217	0	N.E.
**F4**	**C**	**4**	12	0		12	0		103	6	
	**ES**		12	0	N.E.	0	12	0.0001	108	0	0.0291
	**C**	**8**	10	2		12	0		80	23	
	**ES**		4	8	0.0361	5	7	0.0046	93	13	0.0670
	**C**	**10**	4	8		9	3		93	10	
	**ES**		12	0	0.0013	12	0	0.2174	94	12	0.8227
	**C**	**11**	9	2		10	1		77	18	
	**ES**		2	10	0.0033	3	9	0.0028	92	15	0.4461
**F5**	**C**	**6**	16	4		19	1		180	21	
	**ES**		20	0	0.1060	20	0	1.000	202	4	0.0003
	**C**	**8**	18	1		19	0		177	11	
	**ES**		20	0	0.4872	11	9	0.0012	202	5	0.1234
	**C**	**10**	16	4		16	4		213	4	
	**ES**		20	0	0.1060	18	2	0.6614	179	25	0.0001

Solid and slatted floor area was scored on a 5 graded scale, 0 indicating no soiling/blocking. For each score above 0, 25% of the Solid/Slatted floor was considered soiled/blocked. Pig hygiene was scored on a 3 graded scale, 1–3. Score 1 indicate maximum of 20% soiled body surface, score 2; maximum 50% soiled body surface, score 3; more than 50% soiled body surface. Soiling of body surface was scored on one of the pig sides according to Welfare Quality (2009)

*N.E.* non estimable

#### Finishing pig level

In finisher pens, 97.1% of the pigs observed were scored 1 (ranging from 1 to 3), 96.7% in C and 97.4% in ES treatment. On F2, F4 and F5 scores ranged from 1 to 3 in both treatments. On F3 scores ranged from 1 to 3 in C and 1–2 in ES treatment. In total, 0.9% of the observations on F2, 0.8% on F3, 8.1% on F4 and 3.4% on F5 were considered dirty.

Significant treatment effect on pig hygiene was found at three occasions on two of the farms. On F4, the highest straw ratio (ES) had cleaner pigs on occasion 4 (*P* < 0.05). On F5, lowest straw rations (ES) pigs were cleaner on occasion 6 (*P* < 0.001) while C pigs were cleaner on occasion 10 (*P <* 0.001) (Table 1).

### Pen hygiene

#### Solid floor area

Grower level. 91.6% of the C and 96.3% of the ES observations were scored 0 (Table 2). The mean score ranged from 0.0–0.2 on farm level. On Farm G1, G2 and G4, no significant effect of treatment was found on Solid floor hygiene (Table 1).

**Table 2:** Descriptive statistics of pen and pig hygiene scoring for both grower and finishing pigs. C indicates Control treatment and ES Extra Straw treatment. Identical Farm number indicates that the farm produced both grower and finisher pigs

Farm	Treatment	n	Slatted floor area hygiene score						Solid floor area hygiene score							n	Pig hygiene score				
			0	1	2	3	4	median	n	0	1	2	3	4	median		1	2	3	N_**pigs**_	median
	**Growers**																				
	C	164	140	12	8	3	1	0	156	150	13	1	0	0	0	1823	1807	16	0	164	1
	ES	164	142	7	7	0	0	0	156	150	4	2	0	0	0	1743	1735	6	2	161	1
G1		96	94	2	0	0	0	0	96	85	11	0	0	0	0	1149	1140	9	0	96	1
G2		128	93	16	15	3	1	0	128	121	4	3	0	0	0	1350	1335	13	2	133	1
G4		96	95	1	0	0	0	0	96	94	2	0	0	0	0	1067	1067	0	0	96	1
G1	C	48	48	0	0	0	0	0	48	39	9	0	0	0	0	573	567	6	0	48	1
G1	ES	48	46	2	0	0	0	0	48	46	2	0	0	0	0	576	573	3	0	48	1
G2	C	68	45	11	8	3	1	0	68	65	2	1	0	0	0	721	711	10	0	68	1
G2	ES	60	48	5	7	0	0	0	60	56	2	2	0	0	0	629	624	3	2	65	1
G4	C	48	47	1	0	0	0	0	48	46	2	0	0	0	0	529	529	0	0	48	1
G4	ES	48	48	0	0	0	0	0	48	48	0	0	0	0	0	538	538	0	0	48	1
	**Finishers**																				
	C	373	346	23	4	0	0	0	373	332	35	3	1	2	0	3842	3716	95	31	377	1
	ES	352	289	41	15	5	2	0	352	325	22	3	1	1	0	4104	3998	89	17	357	1
F2		233	229	3	1	0	0	0	233	225	8	0	0	0	0	3019	2993	24	2	236	1
F3		95	90	5	0	0	0	0	95	81	13	1	0	0	0	1044	1036	6	2	95	1
F4		159	98	41	14	4	2	0	159	125	25	4	2	3	0	1407	1293	86	28	159	1
F5		238	218	15	4	1	0	0	238	226	11	1	0	0	0	2476	2392	68	16	244	1
F2	C	126	123	2	1	0	0	0	126	120	6	0	0	0	0	1380	1367	12	1	127	1
F2	ES	107	106	1	0	0	0	0	107	105	2	0	0	0	0	1639	1626	12	1	109	1
F3	C	51	49	2	0	0	0	0	51	41	9	1	0	0	0	559	553	4	2	51	1
F3	ES	44	41	3	0	0	0	0	44	40	4	0	0	0	0	482	480	2	0	44	1
F4	C	79	65	13	1	0	0	0	79	66	9	1	1	2	0	695	631	45	19	79	1
F4	ES	80	33	28	13	4	2	0	80	59	16	3	1	1	0	712	662	41	9	80	1
F5	C	117	109	6	2	0	0	0	117	105	11	1	0	0	0	1205	1162	34	9	120	1
F5	ES	121	109	9	2	1	0	1	121	121	0	0	0	0	0	1271	1230	34	7	124	1

Solid and slatted floor area was scored on a 5 graded scale, 0 indicating no soiling/blocking. For each score above 0, 25% of the Solid/Slatted floor was considered soiled/blocked. Pig hygiene was scored on a 3 graded scale, 1–3. Score 1 indicate maximum of 20% soiled body surface, score 2; maximum 50% soiled body surface, score 3; more than 50% soiled body surface. Soiling of body surface was scored on one of the pig sides according to Welfare Quality (2009)

Finishing pig level. 89.4% of the C and 92.0% of the ES observations were scored 0 (Table 2). The mean score was from 0.0–0.4 between the farms. F3 and F2 had no effect of treatment (Table 1). F4, i.e. one of the highest straw rations, C pens were cleaner compared to ES pens on occasion 4 (*p* < 0.001), 8 (*p* < 0.01) and 11 (*p <* 0.01). On Farm F5, i.e. the lowest straw ration, C pens were cleaner compared with ES pens on occasion 8 (*p <* 0.001).

#### Slatted floor area

Grower pig level. 85.4% of the C and 90.7% of the ES observations were scored 0 (Table 2). On G2, i.e. the lowest straw ration, the score ranged from 0 to 4 in C Treatment and 0–2 in ES Treatment. On G2, ES pens were cleaner compared to C pens on occasion 3 (*P* < 0.001), but no treatment effects were seen on farms G1 or G4 (Table 1).

Finishing pig level. 92.5% of the observations in C and 98.4% of the observations in ES were scored 0 (Table 3). The hygiene score ranged between 0 and 2 on F2, 0–1 on F3, 0–4 on F4 and 0–3 on F5. No treatment effect was found on F2, F3 and F5 (Table 2). On F4, C pens were cleaner than ES on occasion 8 (*P* < 0.05) and 11 (*P* < 0.01).

**Table 3:** Production length and number of observations specified per participating farm. Identical Farm number indicates that the farm produced both grower and finisher pigs. WIP indicates weeks in production

Farm	WIP	Number of scorings
G1	5	4
G2	5	3
G5	3	4
F2	13	7
F3	9	5
F4	11	7
F5	10	6

### Correlation between pig and pen hygiene

#### Grower pig level

On Farm level, significant correlations were found on farm G2, where solid hygiene was positively correlated with pig hygiene (*p* < 0.05) and slatted hygiene (*p* < 0.001) (Table 4).

**Table 4:** Correlation between pig hygiene, slatted floor and solid floor hygiene on farm level. The table displays where there was a significant correlation between traits. For the full table, see Appendix I Identical Farm number indicates that the farm produced both grower and finisher pigs. WIP indicates Weeks in Production

Farm	WIP	Pig Hygiene*Slatted floor hygiene	Pig Hygiene*Solid floor hygiene	Solid floor hygiene*Slatted floor hygiene
G2	5	0.11	0.29*	0.44**
F2	5	− 0.10	0.30*	−0.04
	13	−0.08	−0.28	0.46**
F4	8	−0.08	−0.09	0.41*
	10	−0.11	0.15	0.39*
	11	−0.01	0.14	0.49*
F5	2	0.29	−0.05	0.55***
	6	−0.11	0.12	0.48**

*Indicates a *p*-value between 0.01–0.05

**Indicates a *p*-value between 0.001–0.01

***Indicates a *p*-value < 0.001

*N.E.* non estimable

On Treatment level solid and slatted hygiene were highly correlated on farm G1 on WIP (weeks in production) 5 (*p* < 0.0001) (Table 4). In C treatment on G2 Pig hygiene was positively correlated with slatted hygiene (*p* < 0.01) and fully correlated with solid hygiene (*P* > 0.001) and solid and slatted hygiene was positively correlated (*p* < 0.05) on WIP 5. In the ES treatment Solid and slatted hygiene was positively correlated in WIP 5 (*p <* 0.05).

#### Finishing pig level

On Farm level, significant correlations were found on farms F2, F3 and F4 (Table 4). On F2 pig and solid hygiene was positively correlated on WIP 5 (*p* < 0.05) and solid and slatted hygiene on WIP 13 (*p* < 0.001). On F4 positive correlations were found between solid and slatted hygiene on WIP 8 (*p <* 0.05), 10 (*P* < 0.05) and 11 (*p <* 0.05). On F5 positive correlations were found between solid and slatted hygiene on WIP 2(*p <* 0.001) and WIP 6 (*p <* 0.001).

On Treatment level, effects were found on F2, F4 and F5 but not F3. On F2 pig hygiene was positively correlated with slatted hygiene on WIP 5 (*p* < 0.01) WIP 13 (*p <* 0.05) in C treatment (Table 5). In ES treatment solid and slatted hygiene was fully correlated (*p <* 0.001) on F2. On F3 solid and slatted hygiene was positively correlated in WIP 11(*p <* 0.01) in C treatment. On F5 solid and slatted hygiene was positively correlated in WIP 2 (*p <* 0.01) and WIP 6 (*p <* 0.01) in C treatment.

**Table 5:** Correlation between pig hygiene, slatted and solid floor hygiene per farm level on treatment level. The table displays where there was a significant correlation between traits. For the full Table, se Appendix I. Identical Farm number indicates that the farm produces both grower and finisher pigs. WIP indicates Weeks in Production

Farm	WIP	Control			Extra Straw		
		Pig Hygiene * Slatted floor hygiene	Pig hygiene * Solid floor hygiene	Solid floor Hygiene * Slatted floor hygiene	Pig hygiene * Slatted floor hygiene	Pig Hygiene * Solid floor hygiene	Solid floor * Hygiene slatted floor hygiene
G1	5	N.E.	−0.38	N.E.	N.E.	N.E.	1.00***
G2	5	0.40*	1.00***	0.40*	−0.25	−0.11	0.60*
F2	5	−0.11	0.65**	−0.073	N.E.	N.E.	N.E.
	13	−0.09	0.55*	−0.05	− 0.07	−0.07	1.00***
F4	11	0.36	0.34	0.67*	0.05	0.43	0.06
F5	2	0.32	−0.08	0.51*	N.E.	N.E.	N.E.
	6	−0.21	−0.03	0.46*	N.E.	N.E.	N.E.

*Indicates a *p*-value between 0.01–0.05

**Indicates a *p*-value between 0.001–0.01

***Indicates a *p*-value < 0.001

*N.E.* non estimable

## Discussion

Our study did not indicate that pen provided with straw had poor hygiene since the absolute majority of observations were (> 90%) were clean. This experiment was conducted to gain further knowledge on straw impact on pig and pen hygiene in order to facilitate for farmers to make an informed decision regarding functionality of straw in commercial pig production as the compatibility of straw into current production systems, with slatted floors and mechanic manure handling, has been questioned [9, 10].

Whenever there was a Treatment effect on pig hygiene, ES treatment resulted in cleaner pigs, compared C treatment. The effect on pen hygiene was more variable, although most pens were considered clean, and implies that straw has different effects on different farms. One hypothesis is that increased straw rations lead to more straw present on the solid floor making it more distinguished from the slatted area. This might help pigs to differentiate between the lying (solid) and dunging (slatted) areas, leading to less soiling of the lying area and subsequently less soiling of pigs lying in the lying area. On farms with larger C rations, negative effect was mainly seen on the slatted floor hygiene possibly indicating that if larger amounts of straw do end up on the slats it might not pass through the slats and hence reducing slatted floor hygiene. However, it should be noted that over all, the pig and pen hygiene was commonly scored as clean according in all farms.

The limited treatment effects and hygiene effects identified in this study could depend on the relatively small straw rations provided per pen (ranging from ~ 50-600 g/pen/day in C treatment). Previous studies have shown that larger straw rations are needed in order to fully satisfy pigs’ behavioural needs [3, 4] and hence larger straw rations are needed to achieve the welfare expectations that we are aiming for. Larger straw rations might affect hygiene differently and must therefore be investigated further. Already at these levels of straw behaviour and lesions were however affected, and the more straw that the pigs received, the more exploratory behaviour aimed at straw was detected (for further information see 5). The fact that straw provision was interrupted at some occasions on all finishing pig batches (Table 6) might also have affected the results. However, the interruption was equally distributed among Farm 2 and 5 and should therefore have affected both treatments equally. In Farm 3 and 5, ES treatment was interrupted slightly more compared to the C treatment. This might have led to ES treatment being somewhat positively affected, but did not seem to affect the hygiene score in any direction. The farms in this study had different sizes of the straw ration and different length of the straw. It is however difficult to draw conclusions regarding the effect of e.g. straw length on hygiene by comparing the different farms, as they may have other, unknown, characteristics affecting. This, along with the very low variability both within and among farms, makes it difficult to analyse the possible effect of straw length on hygiene. To evaluate such effect, different lengths of straw should be tasted during more controlled circumstances.

**Table 6:** Interruptions in provision of daily straw, % of times. Identical Farm number indicates that the farm produced both grower and finisher pigs. C indicates Control treatment and ES Extra Straw treatment

Grower Farm	C	ES	Finisher Farm	C	ES
*G1*	0	0			
*G2*	0	0	*F2*	11	11
			*F3*	1	6
*G4*	0	0	*F4*	6	6
			*F5*	0.3	0.08

The scoring of pig and pen hygiene was conducted at least 1 h after the daily supervision, when pens were cleaned and provided with straw, which might have affected the results. However, as reported from the same experiment [5], the treatment had no effect on the amount manual cleaning. It could be argued that the pig hygiene is a more longterm effect of the pen hygiene as pigs will get soiled lying in a soiled pen. The pig hygiene will thus sustain also if the pen has been temporarily cleaned. Therefore, the cleanliness of the pigs supports the conclusion that the pen hygiene was good. The low variability in the data made sophisticated modelling of the effect of increased straw difficult, while the descriptive data and simple tests still has great value describing the practicalities of using straw in partly slatted pens.

This study was done on commercial farms, and daily straw provision and reporting was performed by the herdsmen. Studies conducted on-farm differ compared to studies conducted on research farms where research is the main activity. Conducting the experiments in commercial setting in this case meant that we were unable to closely monitor e.g. climate and had to facilitate for farmers to participate in this experiment without obstruct their other work tasks too much. We chose to assign the treatments to facilitate straw provision for the farmers (one row/treatment) instead of randomly assign treatments among pens. This means that we might, involuntary, have grouped pens with the same micro climate within the same treatment, while the other treatment had another microclimate. Pens that clearly had different climatic conditions (such as draft) were excluded to minimize the risk of this, but we were not able to distinguish different micro climates further. However, microclimate such as temperature, humidity or draft is known to affect lying behaviour and hygiene among pigs [12, 13]. The temperature was recorded in connection to the hygiene scoring, one score/batch/occasion. The temperature among batches observed during spring/summer (Finishers Farm 2 and 4) varied more compared to the other seasons. Finishers in Farm 4 had the highest hygiene scores while also having one of the highest standard deviations from the mean temperature, indicating that temperature was unstable during the production period. However, the finishers on Farm 2 experienced similar temperature fluctuations while having one of the lowest hygiene scorings, indicating that this temperature measure is not an accurate indicator by itself. Further, not only air temperature, but factors such as velocity and floor temperature need to be taken into consideration [12, 13]. This could depend on the fact that the fluctuations were of different length in the different farms or were experienced differently among pigs in the different farms or the fact that our measures were to blunt to catch it. This study was not designed to address this further, why this should be taken into consideration in future studies.

In future studies, season as well as micro climatic circumstances should therefore be taken into consideration in order to fully understand the impact of straw (or other bedding material) on hygiene. The accuracy of studies made in commercial settings may therefore be discussed in terms of e.g. controllability and repeatability. In order to increase the reliability the present study, it was repeated on several farms to reduce the impact of farm-specific mistakes. Conducting the experiment in commercial setting aimed to increase the applicability of the results and collect valuable first data within an important subject.

It was impossible to keep farmers or observers blinded to the treatment while conducting this study, which in theory could have affected the results. In order to compensate somewhat for this, the two observers altered between observing the C or ES treatment in order to minimize the impact of the specific observer.

On few occasions, positive correlations were found between solid floor hygiene and pig hygiene, indicating that pigs needed to lie down on a dirty solid area. Correlations between slatted floor hygiene and pig hygiene were less common. Significant correlations between solid and slatted floor pen hygiene were more common, although only found on few occasions. The equivocal results are probably dependant on the low variability and relative cleanliness of the pens, where the possible poor hygiene on the slatted floor is not enough to change the behaviour of the pig to soil also the solid floor and vice versa. Dirty pigs could also be as result of wallowing, i.e. the covering of the body in mud-like substances [14]. The behavioural background of wallowing is not fully understood but is thought to be part of e.g. thermoregulation, grooming, sexual and social behaviour [14]. Wallowing in excreta, the only type of wallowing offered here, has however been advocated as an abnormal behaviour and suggested to happen only when there is no other suitable wallowing substrate available [15, 16]. Further, wallowing in excreta would only serve as thermoregulatory as pigs normally would avoid lying in excreta [15, 16]. Wallowing in excreta is thus rather an indicator of improper environment and not primarily a hygiene issue.

## Conclusions

The amount of straw provided in this study had no effect on the hygiene scoring of the pen floor (solid or slatted) or pen hygiene although previous studies show effect on behaviour and tail lesions. Further, straw could not be inked to large hygiene issues. Pens with poor hygiene were commonly not affected by the amount of straw provided, and hygiene issues are probably also related to other management than the provision of straw. This study did not take important factors such indoor climate (ventilation, humidity, temperature) into further consideration and additional studies regarding these important factors and its relation to pig and pen hygiene are needed in order to draw conclusions.

## Methods

### Animals and housing

The study was conducted in five commercial Swedish pig farms from November 2015 to June 2017. All participation in the study was voluntary, and no compensation was offered to the farmers. All participating farms had commercial pig production in mechanically ventilated and insulated buildings and received veterinary advice regularly according to Swedish regulations. Specific criteria for participation in the study was daily straw provision and partly slatted flooring systems. Due to practical reasons, all farms were located in the south west of Sweden and farms were identified in collaboration with the local farm veterinary advisory service. All pigs were progenies of crossbred sows (Landrace and Yorkshire sows; either Norwegian Landrace*Swedish Yorkshire or Norwegian Landrace*Topigs Large White, that were inseminated with Duroc (G1, G2, F2, F3, G5, F5) or Hampshire boars (F4).

In order to investigate effect of age/size of the pigs, the experiment was conducted on both growers and finishers. One batch each of growing pigs (10 to 30 kg) was investigated on three grower farms: one specialized piglet producing farm (G1), and two farrow-to-finish farms (G2, G4). One batch each of finishing pigs (30 kg – 120 kg) was investigated on four farrow-to finish farms (F2, F3, F4, F5). On F2 and F4 the same batch of pigs was observed in the grower finishing pig stable. The experiment started as the pigs moved in to the grower/finisher unit and finished as the pigs were moved from the grower unit to the first pig was sent to slaughter, respectively. For practical reasons, it was impossible to conduct the experiments at the same period in all farms, and hence the season varied between farms and batches (Table 7). Detailed information about farms and housing is found in Table 8.

**Table 7:** Season and stable temperature at data collection for each farm. Identical farm number within farm id indicates that the farm produced both grower and finisher pigs. The study was performed in Western of Sweden from November 2015 to June 2017

Farm	Age group	Experimental dates	Season	Average temp. ± S.D	Min. temp.	Max. temp.
F1	Growers	9 November-14 December 2015	Autumn, Winter	16.0 ± 2.0	14.0	18.0
F2	Growers	14 March −13 March 2016	Spring	17.3 ± 1.2	16.0	19.0
F4	Growers	23 February- 10 April 2017	Winter, Spring	19.8 ± 0.6	19.0	20.4
F2	Finishers	27 March −19 July 2016	Spring, Summer	23.2 ± 3.0	18.0	27.0
F3	Finishers	22 February – 18 April 2016	Winter, Spring	16.5 ± 0.9	16.0	18.0
F4	Finishers	10 April- 22 June 2017	Spring, Summer	18.4 ± 3.0	15.0	23.1
F5	Finishers	17 February-26 April 2017	Winter, Spring	17.6 ± 1.2	16.7	20.0

Spring: March–May; Summer, June–August; Autumn: September–November; Winter; December–February

**Table 8:** Information on study farms, studied animals and straw provisions. Identical Farm number (within farm id) indicates that the farm produced both grower and finisher pigs. C indicates Control treatment and ES Extra Straw treatment [5]

Farm	Age category	No. pigs produced /year	No. of days in experiment	No. pigs in experiment	No. of pens		Pen Size (m^2^)		No. of pigs / pen	Area/pig (m^2^)	Mean straw length (range).cm	Straw ration. g/pen		Straw ration. g/pig		No. of missing daily obs.
					C	ES	Total	Slatted				C	ES	C	ES	(%)
G1	Growers	18,000	35	286	12	12	4.98	1.19	12	0.41	6.6 (1–40.5)	100 (day 1–15) 200 (day 16–24) 600(day 25–35)	200 500 1000	8.3 16.7 50.0	16.7 41.7 83.3	0 (0)
G2	Growers	7000–7500	34	427	22	17	5.39	1.06	10–11	0.49–0.54	11.9 (7.5–88.5)	47	94	4.3–4.7	8.5–9.4	1 (3)
G4	Growers	7500	43	360	12	12	4.01	1.08	12 (9)^a^	0.33–0.45	5.3 (1–31)	46	92	3.8–5.1^a^	7.6–10.2^a^	2 (5)
F2	Finishers	7000–7500	74	444	21	21	10.49	2.68	11	0.95	11.9 (7.5–88.5)	132	264	12	24	11^b^ (15)
F3	Finishers	2300	70	195	11	9	9.7	1.8	11	0.88	10.4 (1–44)	100	200	9.1	18.2	2 (3)
F4	Finishers	7500	74	209	12	12	9.05	2.25	9	1.00	6.9 (5.5–51.5)	110	220	12.2	24.4	10^c^ (14)
F5	Finishers	10,000	68	408	20	20	10.49	2.68	10	0.95	8.4 (1.5–44.5)	58	115	5.8	11.5	14^d^ (21)

^a^In Farm 4 three pigs per pen were removed after 5 weeks to comply with national legislation according to Swedish board of Agriculture regulations and general advice (SJVFS 2017:25) on the holding of pigs in agriculture

^b^2 weekends and one week

^c^3 weekends

^d^7 weekends

The pigs had daily straw provision (wheat straw, cut in all farms, except Farm 2, see Table 8) and supervision according to normal farm routines. The pigs in each batch were mixed in pens with gilts and castrates and sorted by size; housing the heaviest and the lightest pigs together respectively. Intact litters was practiced when possible, i.e. when the number of pigs in the litter matched the number of places in the pen and the size of the pigs were uniform within the litter. Whenever intact litters were not possible, pigs were sorted by size as described above. All pigs were undocked (according to Swedish legislation). This study was part of a larger study investigating the impact of increased straw ration on pig behaviour and prevalence of tail lesions (see [5]).

### Experimental design and treatments

One batch of pigs, raised within the same physical unit, were studied on each farm. Pens deviating from the average pen regarding pen design or number of pigs were excluded from the study. In Farm 1, only half of the batch was, due to practical reasons, included in the study. In Farm 4 one row of pens in the grower stable was excluded from the study due to deviating climatic circumstances. In Farm 2 and 4 pigs were first studied in the grower stable and then followed into the finishing pig stable.

The studied pens were divided in two equally sized treatments per farm: Control (C), receiving the farm normal straw ration; and Extra Straw (ES), receiving a double C-ration (Table 8). The C-ration was determined and standardized before the experiment by measuring the daily straw ration provided by the herdsmen (Table 8). All pens of the same treatment were located in the same row of the stable unit, to ease for the animal caretaker. Except for straw ration, all pens in the stable unit were on farm level, managed in the same way. Any exceptions from the straw provision routine were, recorded by the caretaker (Table 6). If there was blockage in the slatted flooring, and more than 50% of the slatted area was no longer visible, the daily straw provision could be paused until blockage was cleared.

### Observations

The pig and pen hygiene was scored every second week, including the first and last week of the experiment by two observers (not the farmer) (taking turns scoring C or ES treatment, no intra-observer reliability was measured) (Table 6). The recordings were conducted at least 1 h after the daily cleaning and straw provision.

### Pig hygiene

Pig hygiene was scored according to the Welfare Quality® protocol applied to growing and finishing pigs manure on the body (Welfare Quality 2009). All pigs were individually assessed for manure on the body, on the side that was visible towards the observer according to a three point scale; 1 if a maximum of 20% of the pig was covered in manure; 2: > 20–50% of manure coverage; 3: > 50 of manure coverage.

### Pen hygiene

**Fig. 1:**
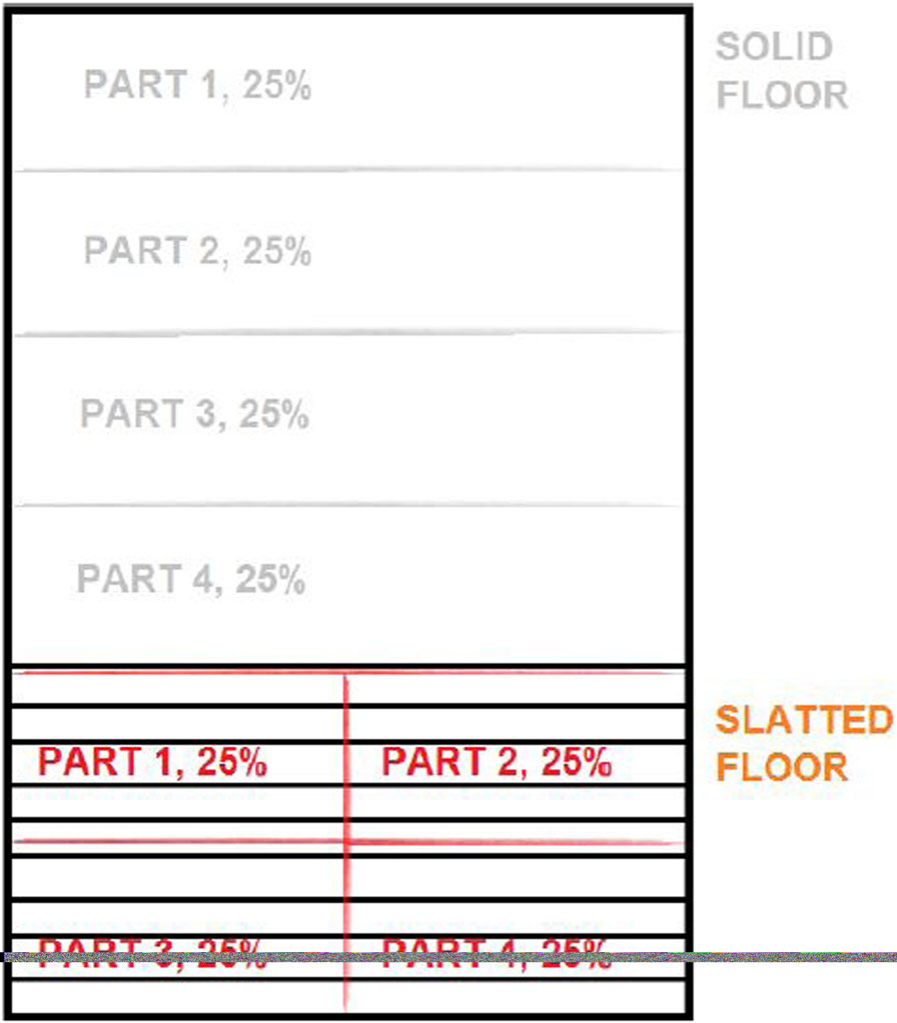
Division of pens into units of assessment

The solid and slatted floor of the pen were each divided in to four separate parts (Fig. 1). The solid floor part was considered dirty when at least 50% of the area was covered by faeces, mired straw or were wet. The slatted floor area was considered as blocked, when the slats was covered and no space between the slats was visible for at least 50% of the assessed area. For each part assessed as dirty/blocked, the pen was scored with one point. Subsequently the scores were added to receive the final hygiene score of the solid and slatted area respectively. A pen could have a maximum of 4 points (all parts ≥50% dirty/blocked) and a minimum of 0 points (all parts < 50% dirty/blocked) per solid/slatted area.

### Statistical analysis

All data were recorded in Microsoft Excel 2016 and analysed through SAS 9.4 (SAS Institute Inc., Cary, NC, USA). The descriptive statistics were calculated through means and frequencies on both farm and age category (grower/finisher) level. Pig and pen hygiene data was ordinal and data had repeated measurements on pen level.

### Pig hygiene

The low variability made analysis of variance on age level impossible. To enable analysis the data was rearranged into binomial traits; hygiene score 1 (i.e. maximum 20% soiled body surface) were considered clean while pigs with score 2–3 (> 20% soiled body surface) were considered dirty. Data was then analysed using Fisher’s exact test, investigating impact of Treatment (C or ES) on Pig hygiene on pig level for each Farm and observation occasion separately.

### Pen hygiene

The low variability made analysis of variance on age level impossible. To enable analysis of variation, the data was rearranged into binomial traits on the solid/slatted floor separately. Pens with the score 0 (no soling/blockage of the floor) were considered clean while pens with score 1–4 (soling/blockage of 25–100% of the Solid/Slatted area) were considered dirty. Data was then analysed using Fisher’s exact test, investigating the impact of Treatment (C or ES) on pen hygiene on pen level for each Farm and observation occasion separately.

### Correlation between pig and pen hygiene

Spearman rank correlation was used to investigate the correlation between pig hygiene and solid/slatted floor hygiene to investigate the hypothesis if pig and pen hygiene was correlated. Pig hygiene was converted into a mean score per pen instead of the initial pig level value. Solid and slatted hygiene was kept as the initial values ranging from 0 to 4 for each occasion. Thus, the correlations were estimated based on pen level scores, for each scoring occasion, both on Farm and Treatment level.

## Supplementary information

**Additional file 1.**

## Data Availability

The datasets used and analysed during the current study are available from the corresponding author on reasonable request.

## Abbreviations

C: Control
ES: Extra Straw

## References

[1] EFSA (2008). EFSA opinion on tail biting and docking in pigs. Vet Rec.

[2] EFSA. Scientific opinion of the Panel on Animal Health and Welfare on a request from Commission on the risks associated with tail biting in pigs and possible means to reduce the need for tail docking considering the different housing and husbandry systems. EFSA J. 2007;611(1–13).

[3] Bodin L, Algers B, Andersson M, Olsson AC, Botermans J (2015). The amount of straw for growing-finishing pigs considering the reduction of time spent in manipulative behavior. SOJ Vet Sci.

[4] Pedersen LJ, Herskin MS, Forkman B, Halekoh U, Kristensen KM, Jensen MB (2014). How much is enough? The amount of straw necessary to satisfy pigs' need to perform exploratory behaviour. Appl Anim Behav Sci.

[5] Wallgren T, Larsen A, Lundeheim N, Westin R, Gunnarsson S (2019). Implication and impact of straw provision on behaviour, lesions and pen hygiene on commercial farms rearing undocked pigs. Appl Anim Behav Sci.

[6] Wallgren T, Westin R, Gunnarsson S (2016). A survey of straw use and tail biting in Swedish pig farms rearing undocked pigs. Acta Vet Scand.

[7] Scott K, Taylor L, Bhupinder PG, Edwards SA (2006). Influence of different types of environmental enrichment on the behaviour of finishing pigs housing in two different systems - 1. Hanging toy versus rootable substrate. Appl Anim Behav Sci.

[8] D'Eath, R.B., Arnott, G., Turner, S.P., Jensen, T., Lahrmann, H.P., Busch, M.E., Niemi, J. K, Lawrence, A.B., Sandøe, P. Injurious tail biting in pigs: how can it be controlled in existing systems without tail docking? Animal, 2014. 8(9): p. 1479–1497. 10.1017/S1751731114001359 25130712

[9] Lahrmann HP, Oxholm LC, Steinmetz H, Nielsen MBF, D’Eathm RBD (2015). The effect of long or chopped straw on pig behaviour. Animal.

[10] Tuyttens FAM (2005). The importance of straw for pig and cattle welfare: a review. Appl Anim Behav Sci.

[11] Scott K, Taylor L, Bhupinder PG, Edwards SA (2007). Influence of different types of environmental enrichment on the behaviour of finishing pigs in two different housing systems - 2. Ratio of pigs to enrichment. Appl Anim Behav Sci.

[12] Olczak K, Nowicki J, Klocek C (2015). Pig behaviour in relation to weather conditions – a review. Ann Anim Sci.

[13] Shi Z, Li B, Zhang X, Wang C, Shou D, Zhang G (2006). Using floor cooling as an approach to improve the thermal environment in the sleeping area in an open pig house. Biosyst Eng.

[14] Bracke MBM (2011). Review of wallowing in pigs: description of the behaviour and its motivational basis. Appl Anim Behav Sci.

[15] Bracke MBM, Spoolder HAM (2011). Review of wallowing in pigs: implications for animal welfare. Anim Welf.

[16] Huynh TTT, Aarnink AJA, Gerrits WJJ, Heetkamp MJH, Canh TT, Spoolder HAM, Kemp B, Verstegen MWA (2005). Thermal behaviour of growing pigs in response to high temperature and humidity. Appl Anim Behav Sci.

[17] The Swedish board of Agriculture’s regulations and general guidelines for experimental animals. 2019 SJVFS 2019:9 2 Chapt. 18 §.

