# Supplementary material for: Impact of amount of straw on pig and pen hygiene in partly slatted flooring systems
Source: BMC Vet Res. 2020 Oct 7;16:377. doi: 10.1186/s12917-020-02594-y (PMC7542705; doi:10.1186/s12917-020-02594-y)
Supplement: Supplementary file 1 — Additional file 1. [file 12917_2020_2594_MOESM1_ESM.docx]

Appendix Table 4. The full output from Table 4. Impact of treatment on pig and pen hygiene scores.

|  |  |  | **Slatted Hygiene** | | | **Solid Hygiene** | | | **Pig Hygiene** | | | |  |
| --- | --- | --- | --- | --- | --- | --- | --- | --- | --- | --- | --- | --- | --- |
| **Farm** | **Treatment** | **WIP** | **Clean** | **Not Clean** | **p-value** | **Clean** | **Not Clean** | **p-value** | | **Clean** | **Not Clean** | **p-value** | |
| **G1** | **C** | **1** | 12 | 0 |  | 12 | 0 |  | | 144 | 0 |  | |
|  | **ES** |  | 12 | 0 | N.E. | 12 | 0 | N.E. | | 144 | 0 | N.E. | |
|  | **C** | **3** | 12 | 0 |  | 9 | 3 |  | | 144 | 0 |  | |
|  | **ES** |  | 11 | 1 | 1.000 | 12 | 0 | 0.2174 | | 144 | 0 | N.E. | |
|  | **C** | **4** | 12 | 0 |  | 11 | 1 |  | | 141 | 3 |  | |
|  | **ES** |  | 12 | 0 | N.E. | 11 | 1 | 1.000 | | 141 | 3 | 1.000 | |
|  | **C** | **5** | 12 | 0 |  | 7 | 11 |  | | 138 | 3 |  | |
|  | **ES** |  | 11 | 1 | 1.000 | 5 | 1 | 0.1550 | | 144 | 0 | 0.1198 | |
| **G2** | **C** | **1** | 16 | 4 |  | 18 | 1 |  | | 217 | 0 |  | |
|  | **ES** |  | 20 | 0 | 0.1060 | 20 | 0 | 0.4872 | | 200 | 0 | N.E. | |
|  | **C** | **3** | 14 | 10 |  | 24 | 0 |  | | 253 | 0 |  | |
|  | **ES** |  | 20 | 0 | 0.0009 | 20 | 0 | N.E. | | 217 | 0 | N.E. | |
|  | **C** | **5** | 15 | 9 |  | 23 | 1 |  | | 241 | 10 |  | |
|  | **ES** |  | 8 | 12 | 0.2253 | 16 | 4 | 0.1605 | | 207 | 5 | 0.4321 | |
| **G4** | **C** | **1** | 12 | 0 |  | 12 | 0 |  | | 144 | 0 |  | |
|  | **ES** |  | 12 | 0 | N.E. | 12 | 0 | N.E. | | 144 | 0 | N.E. | |
|  | **C** | **2** | 12 | 0 |  | 10 | 2 |  | | 137 | 0 |  | |
|  | **ES** |  | 12 | 0 | N.E. | 12 | 0 | 0.4783 | | 143 | 0 | N.E. | |
|  | **C** | **4** | 12 | 0 |  | 12 | 0 |  | | 140 | 0 |  | |
|  | **ES** |  | 12 | 0 | N.E. | 12 | 0 | N.E. | | 143 | 0 | N.E. | |
|  | **C** | **6** | 11 | 1 |  | 12 | 0 |  | | 108 | 0 |  | |
|  | **ES** |  | 12 | 0 | 1.000 | 12 | 0 | N.E. | | 108 | 0 | N.E. | |
| **F2** | **C** | **1** | 21 | 0 |  | 21 | 0 |  | | 231 | 0 |  | |
|  | **ES** |  | 20 | 1 | 1.000 | 21 | 0 | N.E. | | 231 | 0 | N.E. | |
|  | **C** | **3** |  |  |  |  |  |  | |  |  |  | |
|  | **ES** |  |  |  |  |  |  |  | | 231 | 0 | N.E. | |
|  | **C** | **5** | 20 | 1 |  | 19 | 2 |  | | 227 | 3 |  | |
|  | **ES** |  | 21 | 0 | 1.000 | 21 | 0 | 0.4878 | | 221 | 7 | 0.2199 | |
|  | **C** | **7** | 21 | 0 |  | 21 | 0 |  | | 227 | 0 |  | |
|  | **ES** |  | 21 | 0 | N.E. | 21 | 0 | N.E. | | 228 | 1 | 1.000 | |
|  | **C** | **10** | 20 | 1 |  | 21 | 0 |  | | 223 | 3 |  | |
|  | **ES** |  | 21 | 0 | 1.000 | 21 | 0 | N.E. | | 449 | 3 | 0.4059 | |
|  | **C** | **11** | 18 | 3 |  | 21 | 0 |  | | 225 | 0 |  | |
|  | **ES** |  | 2 | 0 | 1.000 | 2 | 0 | N.E. | | 23 | 0 | N.E. | |
|  | **C** | **13** | 20 | 1 |  | 20 | 1 |  | | 234 | 7 |  | |
|  | **ES** |  | 20 | 1 | 1.000 | 20 | 1 | 1.000 | | 227 | 2 | 0.1768 | |
| **F3** | **C** | **1** | 6 | 7 |  | 8 | 0 |  | | 88 | 0 |  | |
|  | **ES** |  | 2 | 1 | 1.000 | 8 | 0 | N.E. | | 88 | 0 | N.E. | |
|  | **C** | **4** | 9 | 2 |  | 9 | 2 |  | | 122 | 0 |  | |
|  | **ES** |  | 7 | 2 | 1.000 | 8 | 1 | 1.000 | | 99 | 0 | N.E. | |
|  | **C** | **5** | 9 | 2 |  | 11 | 0 |  | | 122 | 0 |  | |
|  | **ES** |  | 8 | 0 | 0.4854 | 8 | 0 | N.E. | | 88 | 0 | N.E. | |
|  | **C** | **7** | 10 | 1 |  | 11 | 0 |  | | 116 | 6 |  | |
|  | **ES** |  | 9 | 0 | 1.000 | 7 | 2 | 0.1895 | | 96 | 2 | 0.3038 | |
|  | **C** | **9** | 7 | 3 |  | 10 | 0 |  | | 108 | 0 |  | |
|  | **ES** |  | 9 | 1 | 0.5820 | 10 | 0 | N.E. | | 109 | 0 | N.E. | |
| **F4** | **C** | **1** | 8 | 0 |  | 8 | 0 |  | | 72 | 0 |  | |
|  | **ES** |  | 6 | 2 | 0.4667 | 7 | 1 | 1.000 | | 71 |  | 1.000 | |
|  | **C** | **2** | 12 | 0 |  | 5 | 7 |  | | 108 | 0 |  | |
|  | **ES** |  | 12 | 0 | N.E. | 1 | 11 | 0.1550 | | 108 | 0 | N.E. | |
|  | **C** | **4** | 12 | 0 |  | 12 | 0 |  | | 103 | 6 |  | |
|  | **ES** |  | 12 | 0 | N.E. | 0 | 12 | 0.0001 | | 108 | 0 | 0.0291 | |
|  | **C** | **6** | 11 | 1 |  | 9 | 3 |  | | 98 | 7 |  | |
|  | **ES** |  | 11 | 1 | 1.000 | 5 | 7 | 0.2138 | | 96 | 9 | 0.7957 | |
|  | **C** | **8** | 10 | 2 |  | 12 | 0 |  | | 80 | 23 |  | |
|  | **ES** |  | 4 | 8 | 0.0361 | 5 | 7 | 0.0046 | | 93 | 13 | 0.0670 | |
|  | **C** | **10** | 4 | 8 |  | 9 | 3 |  | | 93 | 10 |  | |
|  | **ES** |  | 12 | 0 | 0.0013 | 12 | 0 | 0.2174 | | 94 | 12 | 0.8227 | |
|  | **C** | **11** | 9 | 2 |  | 10 | 1 |  | | 77 | 18 |  | |
|  | **ES** |  | 2 | 10 | 0.0033 | 3 | 9 | 0.0028 | | 92 | 15 | 0.4461 | |
| **F5** | **C** | **1** | 18 | 0 |  | 18 | 0 |  | | 195 | 0 |  | |
|  | **ES** |  | 20 | 0 | N.E. | 20 | 0 | N.E. N.E. | | 227 | 0 | N.E. | |
|  | **C** | **2** | 19 | 1 |  | 17 | 3 |  | | 200 | 2 |  | |
|  | **ES** |  | 20 | 0 | 1.000 | 20 | 0 | 0.2308 | | 208 | 1 | 0.6178 | |
|  | **C** | **4** | 18 | 2 |  | 20 | 0 |  | | 197 | 5 |  | |
|  | **ES** |  | 21 | 0 | 0.2317 | 20 | 1 | 1.000 | | 212 | 6 | 1.000 | |
|  | **C** | **6** | 16 | 4 |  | 19 | 1 |  | | 180 | 21 |  | |
|  | **ES** |  | 20 | 0 | 0.1060 | 20 | 0 | 1.000 | | 202 | 4 | 0.0003 | |
|  | **C** | **8** | 18 | 1 |  | 19 | 0 |  | | 177 | 11 |  | |
|  | **ES** |  | 20 | 0 | 0.4872 | 11 | 9 | 0.0012 | | 202 | 5 | 0.1234 | |
|  | **C** | **10** | 16 | 4 |  | 16 | 4 |  | | 213 | 4 |  | |
|  | **ES** |  | 20 | 0 | 0,1060 | 18 | 2 | 0,6614 | | 179 | 25 | 0,0001 | |

Solid and slatted floor area was scored on a 5 graded scale, 0 indicating no soiling/blocking. For each score above 0, 25% of the Solid/Slatted floor was considered soiled/blocked. Pig hygiene was scored on a 3 graded scale, 1-3. Score 1 indicate maximum of 20% soiled body surface, score 2; maximum 50% soiled body surface, score 3; more than 50% soiled body surface. Soiling of body surface was scored on one of the pig sides according to Welfare Quality (2009). N.E.: non estimable.

Missing data for F2 Treatment ES WIP 3 and 11.

Appendix Table 5. Full Table 5. Correlation between pig hygiene, slatted and solid pen hygiene on farm level. The table displays where there was a significant correlation between traits.

| **Farm** | **WIP** | **Pig Hygiene*Slatted Hygiene** | **Pig Hygiene*Solid Hygiene** | **Solid Hygiene*Slatted Hygiene** |
| --- | --- | --- | --- | --- |
| G1 | 1 | N.E. | N.E. | N.E. |
|  | 3 | N.E. | N.E. | -0.08 |
|  | 4 | N.E. | 0.52 | N.E. |
|  | 5 | -0.62 | -0.17 | 0.36 |
| G2 | 1 | N.E. | N.E. | -0.08 |
|  | 3 | N.E. | N.E. | N.E. |
|  | 5 | 0.11 | 0.29* | 0.44** |
| G4 | 1 | N.E. | N.E. | N.E. |
|  | 2 | N.E. | N.E. | N.E. |
|  | 4 | N.E. | N.E. | N.E. |
|  | 6 | N.E. | N.E. | N.E. |
| F2 | 1 | N.E. | N.E. | N.E. |
|  | 3 | Missing | Missing | Missing |
|  | 5 | -0.10 | 0.30* | -0.04 |
|  | 7 | N.E. | N.E. | N.E. |
|  | 10 | N.E. | -0.04 | N.E. |
|  | 11 | N.E. | N.E. | N.E. |
|  | 13 | -0.08 | -0.28 | 0.46** |
| F3 | 1 | N.E. | N.E. | N.E. |
|  | 3 | N.E. | N.E. | 0.14 |
|  | 5 | N.E. | N.E. | N.E. |
|  | 7 | -0.24 | 0.24 | -0.08 |
|  | 9 | N.E. | N.E. | N.E. |
| F4 | 1 | -0.07 | -0.1 | -0.1 |
|  | 2 | N.E. | N.E. | N.E. |
|  | 4 | -0.28 | N.E. | N.E. |
|  | 6 | -0.11 | -0.26 | 0.05 |
|  | 8 | -0.08 | -0.09 | 0.41* |
|  | 10 | -0.11 | 0.15 | 0.39* |
|  | 11 | -0.01 | 0.14 | 0.49* |
| F5 | 1 | N.E. | N.E. | N.E. |
|  | 2 | 0.29 | -0.05 | 0.55*** |
|  | 4 | -0.08 | -0.11 | -0.04 |
|  | 6 | -0.11 | 0.12 | 0.48** |
|  | 8 | -0.13 | -0.11 | -0.09 |
|  | 10 | -0.13 | -0.32* | -0.14 |
|  |  |  |  |  |

N.E.: non estimable.

Table 6. Full Table 6. Correlation between pig hygiene, slatted and solid pen hygiene per farm level on treatment level.

| **Farm** | **WIP** | **Control** | | | **Extra Straw** | | |
| --- | --- | --- | --- | --- | --- | --- | --- |
|  |  | **Pig Hygiene Slatted Hygiene** | **Pig Hygiene Solid Hygiene** | **Solid Hygiene Slatted Hygiene** | **Pig Hygiene Slatted Hygiene** | **Pig Hygiene Solid Hygiene** | **Solid Hygiene Slatted Hygiene** |
| G1 | 1 | N.E. | N.E. | N.E. | N.E. | N.E. | N.E. |
|  | 3 | N.E. | N.E. | N.E. | N.E. | N.E. | N.E. |
|  | 4 | N.E. | 0.52 | N.E. | N.E. | 0.52 | N.E. |
|  | 5 | N.E. | -0.38 | N.E. | N.E. | N.E. | 1.00*** |
| G2 | 1 | N.E. | N.E. | -0.17 | N.E. | N.E. | N.E. |
|  | 3 | N.E. | N.E. | N.E. | N.E. | N.E. | N.E. |
|  | 5 | 0.40* | 1.00*** | 0.40* | -0.25 | -0.11 | 0.60* |
| G4 | 1 | N.E. | N.E. | N.E. | N.E. | N.E. | N.E. |
|  | 2 | N.E. | N.E. | N.E. | N.E. | N.E. | N.E. |
|  | 4 | N.E. | N.E. | N.E. | N.E. | N.E. | N.E. |
|  | 6 | N.E. | N.E. | N.E. | N.E. | N.E. | N.E. |
| F2 | 1 | N.E. | N.E. | N.E. | N.E. | N.E. | N.E. |
|  | 3 | N.E. | N.E. | N.E. | Missing | Missing | Missing |
|  | 5 | -0.11 | 0.65** | -0.073 | N.E. | N.E. | N.E. |
|  | 7 | N.E. | N.E. | N.E. | N.E. | N.E. | N.E. |
|  | 10 | N.E. | -0.07 | N.E. | N.E. | N.E. | N.E. |
|  | 11 | N.E. | N.E. | N.E. | N.E. | N.E. | N.E. |
|  | 13 | -0.09 | 0.55* | -0.05 | -0.07 | -0.07 | 1.00*** |
| F3 | 1 | N.E. | N.E. | N.E. | N.E. | N.E. | N.E. |
|  | 4 | N.E. | N.E. | 0.39 | N.E. | N.E. | -0.19 |
|  | 5 | N.E. | N.E. | N.E. | N.E. | N.E. | N.E. |
|  | 7 | N.E. | 0.17 | N.E. | -0.29 | N.E. | N.E. |
|  | 9 | N.E. | N.E. | N.E. | N.E. | N.E. | N.E. |
| F4 | 1 | N.E. | N.E. | N.E. | -0.14 | -0.22 | -0.22 |
|  | 2 | N.E. | N.E. | N.E. | N.E. | N.E. | N.E. |
|  | 4 | N.E. | N.E. | N.E. | N.E. | N.E. | N.E. |
|  | 6 | 0.00 | -0.24 | -0.17 | -0.25 | -0.29 | 0.19 |
|  | 8 | N.E. | 0.31 | N.E. | 0.31 | -0.14 | 0.34 |
|  | 10 | -0.15 | 0.36 | 0.35 | N.E. | N.E. | N.E. |
|  | 11 | 0.36 | 0.34 | 0.67* | 0.05 | 0.43 | 0.06 |
| F5 | 1 | N.E. | N.E. | N.E. | N.E. | N.E. | N.E. |
|  | 2 | 0.32 | -0.08 | 0.51* | N.E. | N.E. | N.E. |
|  | 4 | N.E. | -0.17 | N.E. | -0.11 | N.E. | N.E. |
|  | 6 | -0.21 | -0.03 | 0.46* | N.E. | N.E. | N.E. |
|  | 8 | N.E. | -0.17 | N.E. | -0.09 | N.E. | N.E. |
|  | 10 | -0.21 | -0.21 | -0.25 | 0.10 | N.E. | N.E. |

*Indicates a p-value between 0.01-0.05
**Indicates a p-value between 0.001-0.01

***Indicates a p-value <0.001

N.E.: non estimable.
